# Prophylactic Inoculations

**Published:** 1899-12

**Authors:** Edward F. Willoughby

**Affiliations:** London, England


### Abstracts and Translations.

### PROPHYLACTIC INOCULATIONS.

BY EDWARD F. WILLOUGHBY, M.D., LONDON, ENGLAND.

THE progress of bacteriology during the last few years has been so rapid that it is almost impossible for any but those who are actually engaged in the work of the laboratory to keep abreast of the advance of the science. Working hypotheses either become established facts or are set aside by the very discoveries they have led up to, almost before their true character has been generally apprehended. One result of this advance all along the line is that the public as represented by the lay press, and a large part, probably the majority, of the medical profession, when the latest discoveries find a practical application in the prevention or treatment of disease, are apt to interpret them in the light of the state of the science and the knowledge of several years past, and to ascribe to particular procedures the characters and effects of others more familiar to them, but with which they have little or nothing in common. They are fully aware of the permanent or, at any rate, long duration of the protection against small-pox conferred by vaccination, and probably conclude that inoculations against cholera or diphtheria must be equally lasting; or, finding that the immunity is only temporary and transient, are inclined to doubt its reality altogether. They do not clearly distinguish between immunization by the induction of the actual disease in an attenuated or modified form, and that imparted by the injection of products of the bacilli, which, so far from producing the phenomena of the disease in a milder phase, have a directly opposite and antagonistic action; and fewer still have any conception of the difference between antibiotic and antitoxic agents, the one class causing the death of the bacilli, and the other neutralizing or destroying the poison independently of any action they may or may not exert on the bacilli themselves. There is, in fact, nothing improbable in the statement that the bacilli of diphtheria have been cultivated in the (solution of) antitoxin, though to such men it would appear to prove the whole doctrine of sero-therapy a delusion; and the fact, well known to experts, that quantities of (washed) cultures of tetanus bacilli may be introduced into a (healthy) wound, but that the addition of a little lactic acid or of a culture of bacillus prodigiosus will induce tetanic phenomena, would seem to prove that these were due to the accessories rather than to the reputed specific microbe; and, lastly, the indisputable fact that many persons in seeming health carry the bacillus of diphtheria in their mouths for weeks together without any infection or ill-effects to themselves is in itself calculated to provoke incredulity as to the existence of an essential and strictly causal relation, and to suggest a merely casual connection or association between the bacillus and the disease.

At the present time this confusion of conception is prevalent as regards the preventive and curative treatment of the plague in India. Because the serum employed by Yersin and Haffkine has yielded results very far from satisfactory, if not really negative, little credit is given to that employed by Drs. Galeotti and Polverini, though it is, in fact, a totally different preparation, being an antitoxin, while the others are, or were, since their use has been discontinued, "vaccines" or attenuations of the actual virus, in accordance with the practice of Pasteur, whereas the Italian serum is, or contains, a substance strictly analogous to the antitoxins of diphtheria and tetanus which we owe to Behring, Kitasato, and Cantani.

The products of bacteria by which the phenomena of infection and of immunity are induced belong to two distinct classes, ptomaines and toxines; the former being alkaloids, of complex but definite composition like those obtained from the vegetable world, capable of being isolated in a state of purity, and secondly of albumoses, akin to those produced in the process of peptic and tryptic digestion, of the chemical constitution of which little is known, from the impossibility of isolating them from the inert albuminoids in and with which they are held in solution, and from their tendency to the total or partial loss of their properties in the act of drying. An interesting fact may be here mentioned in passing,—viz., that the venom of serpents, which is an albumen or an albumose, though a secretion of the parotid gland and not a product of bacteria, is susceptible, on the one hand, of being digested by the action of ptyalin, papain, pancreatin, and pepsin, so as to be rendered inert when swallowed, and on the other, is capable of acting as a peptonizing agent on other albuminoids, etc.

Ptomaines and toxines are alike poisonous, and alike vary greatly in their virulence; but ptomaines are exclusively products of the bacteria of putrefaction, under circumstances involving the exclusion or a very scanty supply of oxygen. The virulence of some almost passes belief, while others are scarcely poisonous in the ordinary meaning of the word. The toxines are for the most part less virulent than the ptomaines, that of tetanus being the most, and those of diphtheria, and probably the plague, coming next in their virulence. The power possessed by many bacteria of dissolving and digesting animal matter plays probably an important part in their action on the living body. Of one hundred and forty kinds observed by Claudio Fermi, forty liquefied gelatin and five dissolved fibrin, while a large number, though cultivated in serum, gelatin, and broths, in the entire absence of starch or sugar, yielded diastatic ferments; those of anthrax, cholera, and Finkler and Prior's being among the number. But while neither class of ferments is formed by bacteria grown in nutrient *salts* only, the presence of starch is *most favorable* to the formation of diastatic ferments, and of gelatin to that of the peptonizing, the conditions of temperature, reaction, etc., being within the limits requisite for each particular case.

Some of the poisons produced by bacteria act simply as do the vegetable alkaloids, especially muscarine, as the gland poison of snakes, or induce indefinite symptoms of gastro-intestinal irritation. Others are pyogenic, exciting local inflammation, with the formation of pus. Among these the general tendency of streptococcal inflammation is to a wide diffusion of the suppuration, while that of the staphylococcal is to circumscribed abscesses, the process being limited by the fibrinous exudation peculiar to their action. In like manner, the density of the so-called false membrane in diphtheria will "vary from the consistence of cream to that of wash-leather," just as streptococci or staphylococci predominate, Löffler's bacillus, which alone induces the remote effects, the degeneration of the medullary axes of nerve-fibres, and of the muscular tissue of the heart, taking little or no part in the exudation.

A distinction has been made between toxic and septic poisons, the former being those produced locally by the bacteria, and absorbed and diffused thence by the lymphatics; the latter being formed everywhere by the bacteria, which, multiplying in the blood, pervade the entire circulation and penetrate the tissues, instead of remaining confined to certain glands or organs, as the spleen. The distinction, however, is somewhat artificial, some bacteria producing one or other poison under different conditions, or even apparently both under the same.

Immunity is the power possessed or acquired by the organism of producing or causing to be produced substances having (1) the property of causing the death or of inhibiting the growth of the special pathogenic bacteria, or (2) of destroying or of neutralizing the action of the toxines; and immunity of either kind may be (1) natural or congenital to the race or the individual, or (2) may be acquired by the animal having passed through an attack of the disease or having been subjected to certain processes of inoculation with prophylactic preparations; and the immunity of either kind and whensoever derived may be but transient and temporary or so far permanent as to be practically of lifelong duration.

Natural immunity is possessed by certain animals towards certain diseases, sometimes in virtue of the high or low temperature of their blood,[1] in others it is as inexplicable as their tolerance of morphine, atropine, etc., the resistance being manifested in some towards the growth of the bacilli, the tolerance in others in respect of the toxines.

Acquired immunity, temporary or permanent, follows an attack of certain diseases, small-pox, scarlatina, measles, typhus, and yellow fever conferring a lifelong exemption, and diphtheria one of little more than a month's duration. But it may be imparted artificially by inducing a modified form of the disease, as in vaccination, the vaccine virus being not that of a disease peculiar to the cow, but simply variolous virus so altered by cultivation in the organism of a bovine or equine animal to which it is foreign as to have lost most of its virulence, and becomes communicable by actual inoculation only, enthetic instead of contagious; or by the attenuation of the virus by cultivation under unfavorable conditions, as in

---

[1] The immunity of birds to anthrax can be overcome by lowering their temperature, and in fish the bacillus of tubercle induces a rapidly fatal disease, but loses its virulence towards warm-blooded animals.

the living bodies of other animals, or in culture fluids, or at temperatures partially inhibiting the normal development and vital functions of the bacteria. Such are the Pasteurian inoculations against anthrax, rabies, rouget, etc. But from the impossibility of accurately standardizing the cultures, and the differing susceptibilities of individual animals, there is always the risk of either failing to obtain the requisite degree of resistance, or of giving a dose that shall prove fatal; so that, however useful with brutes, whose lives have only a pecuniary value, the risks attending these inoculations preclude their employment in the case of man. Immunity may also be conferred by injections of the toxines, but it is not so lasting as that following injections of living bacteria, and requires a certain time for its acquisition. It is easier to render an animal immune to fatal doses of living bacteria than to those of their toxines, and the former immunity does not necessarily involve the latter, though the latter does the former.

There is one more way in which immunity may be imparted,—viz., the injection of the serum of animals highly immunized by other means. Of this the antitoxin treatment of diphtheria is the type, that of tetanus and the Italian method of treating the plague being the same in principle, though they have not as yet attained equal certainty and accuracy. This procedure, consisting in the injection not of the toxine, but of the antitoxin, not of the disease in even the most attenuated form, but of its very opposite, possesses advantages over every other, and being conceivably available in respect of every specific disease that of itself tends to a spontaneous termination within a definite period, and of all those that confer immunity of longer or shorter duration, holds out prospects as to the prevention of infection and the arrest or cure of disease, the importance of which it is scarcely possible as yet to estimate.

The resistance of an animal to infection is a complex effort involving phagocytosis, or the action of the leucocytes on the bacteria, towards which they are attracted by the phenomenon of chemiotaxis, and which they take into their substance and devour as an amœba does the particles on which it feeds; a process which takes place in every exposure to infection, the struggle ending in favor of the leucocytes, or of the bacteria. It is thus that a man, who after having long resisted exposure to infection, as in a fever hospital, at length succumbs, when by fatigue, hunger, or any depressing circumstance the vitality and energy of his protoplasm has been lowered. The serum in its normal state is possessed of a certain bactericidal property, in virtue, probably, of some substances formed by the leucocytes, called alexins, though their existence is at present only hypothetical.

But the paramount means by which the disease is brought to a termination is the production of an antitoxin, the property of which is to act as an antidote neutralizing the toxine secreted by the bacilli, which are meanwhile destroyed by phagocytosis, or gradually succumb to the bactericidal power of the serum. The protoplasm of an animal that has gone through a natural attack of a disease contains such antitoxin, but in quantities too small for practical purposes, for which such a high degree of immunity as can be obtained only by long-continued artificial immunization is necessary. The horse, though not insusceptible to the poison of diphtheria, possesses the power of producing the antitoxin in so extraordinary a degree that it is almost impossible to induce the fully developed disease in him, except by using enormous quantities of the toxine. The procedure originated by Behring consists in the injection of a certain quantity of a highly virulent culture of the diphtheria bacillus that has been carried on for a month at the temperature of the blood. This gives rise to a febrile disturbance of short duration, and so soon as it has subsided the injection is repeated, with little, if any, visible result. Gradually increasing doses are injected at short intervals till, at the end of three months, doses several hundred times as great as those first used can be injected with absolutely no effect on the health or well-being of the animal, whose blood is then so charged with antitoxin that very small quantities of its serum are required to confer immunity on or to arrest the progress of the disease in any other animal susceptible to it. Henceforth occasional injections suffice to maintain the condition of the horse's blood, and a litre or more may be drawn off every week. The serum, separated by the coagulation of the fibrin and red blood-corpuscles, carefully filtered and bottled with antiseptic precautions, constitutes the antitoxine so called, or, more correctly, is a solution of the antitoxin, the isolation of which in the dried state has not yet been very successful. The "antitoxin" is standardized by determining the quantity capable of exactly neutralizing the minimum lethal dose of toxine required by a guinea-pig of average size.

If administered in *sufficient quantity* to a patient within the first twenty-four hours from the commencement of the illness, the mortality may be reduced to one per cent., and if within three days to five per cent. At later stages its effect becomes less and less marked; indeed, after the seventh day it has little influence, for, though it may avert further destruction of the nerve-substance and the fibres of the cardiac muscles, it is obvious that it cannot undo the degenerative processes that have already taken place.

It may also be used for prophylaxis, one good injection rendering the individual insusceptible of infection for a period of, but not exceeding, one month. This use of antitoxin has not been taken advantage of as extensively as it should be. On the appearance of a case of diphtheria in a family or school, it should be employed not only on the actual patient in whom the disease may perhaps have already proceeded too far for much effect, but on every other child in the establishment, and adults in attendance on the patient. They will thus be protected for a period long enough to cover the whole course of the original case, and the disinfection of the room and all possible vehicles of contagion.

Much has been written of late about antistreptococcal serums, but their efficacy is far from proven, and there is no satisfactory evidence that they are in any way comparable, the virus of septicæmia, etc., belonging to the class of septic rather than of toxic substances.

It is generally held that the antitoxins are secreted by the protoplasm of the cells, and not by the bacteria; but since it is not easy to believe that these cells have the power of producing a separate antitoxin for every possible toxine, it seems a more reasonable hypothesis to ascribe to them the property of acting on the toxine itself in such a manner as to obtain from it or to convert it into an antitoxin; and experiments *in vitro* on certain toxines appear to give support to this view of the mutual relations of the cells, the toxines, and the antitoxins.—*The Therapist.*

